# Adverse effects of remdesivir for the treatment of acute COVID-19 in the pediatric population: a retrospective observational study

**DOI:** 10.1186/s40348-024-00175-9

**Published:** 2024-02-21

**Authors:** Abigail Schulz, Natalie Huynh, Margaret Heger, Mustafa Bakir

**Affiliations:** 1 University of Illinois College of Medicine at Peoria, 530 NE Glen Oak Avenue, North Building #6606, Peoria, IL 61637 USA; 2 Department of Pediatrics, University of Illinois College of Medicine at Peoria, 530 NE Glen Oak Avenue, North Building #6606, Peoria, IL 61637 USA; 3 Department of Pharmacy, OSF HealthCare Children’s Hospital of Illinois, Peoria, IL USA; 4 Department of Pediatrics, Division of Pediatric Infectious Diseases, University of Illinois College of Medicine at Peoria, 530 NE Glen Oak Avenue, North Building #6606, Peoria, IL 61637 USA

**Keywords:** Remdesivir, Adverse effects, Children

## Abstract

**Background:**

Although the severity of coronavirus disease 2019 (COVID-19) tends to be lower in children, it can still lead to severe illness, particularly among those with chronic medical conditions. While remdesivir (RDV) is one of the few approved antiviral treatments for COVID-19 in children in many countries, the available data on the safety of RDV in this population is limited.

**Methods:**

To address this knowledge gap, a multicenter study involving 65 patients retrospectively analyzed the clinical data from individuals aged <18 who were hospitalized due to severe COVID-19 (defined as SpO_2_ < 94% or requiring supplemental oxygen) and received at least one dose of RDV. Additionally, the study encompassed 22 patients with mild-moderate COVID-19 who were considered at high risk of developing severe disease.

**Results:**

Nineteen children (29%) experienced mild-to-moderate adverse events (AEs) attributed to RDV, including transaminitis in 20% of children, bradycardia in 8%, and hypotension in 5%. AEs did not require discontinuation of RDV, except in one patient who developed premature ventricular contractions. The rate of AEs did not differ between patients with severe COVID-19 and those with mild-moderate COVID-19 but at high risk for severe disease. All but one patient were discharged within 23 days of admission, and no fatalities were recorded. Among high-risk patients with mild-moderate disease, only 2 (9%) progressed to the point of needing supplemental oxygen.

**Conclusions:**

Our data suggests that RDV is safe in children, with no reported serious AEs. However, the absence of a control group limits the extent to which conclusions can be drawn. RDV may contribute to clinical improvement, particularly in high-risk patients.

## Background

Over 17 million cases of coronavirus disease 2019 (COVID-19) in children have been recorded in the United States (U.S.) as of September 2023 [1]. Compared to adults, COVID-19 generally has a less severe course in the pediatric population and does not commonly require specific treatment, with individuals under 18 years old comprising approximately 0.4% of COVID-19-related deaths in the U.S. [1]. Despite this, some children can progress to develop critical illness, including complications such as acute respiratory distress syndrome (ARDS) or multisystem inflammatory syndrome in children (MIS-C) [2, 3]. Children with obesity, complex chronic medical conditions, and ventilator or gastrostomy tube dependence are particularly at high risk for severe COVID-19. Racial disparities in COVID-19 morbidity and mortality are also well-established, with severe COVID-19 disproportionately impacting children of Hispanic and African American race [4, 5]. Thus, safe and effective therapeutics become even more imperative for these vulnerable, high-risk children.

Remdesivir (RDV) (Veklury®, Gilead Sciences) is an adenosine analogue prodrug that inhibits transcription in a broad range of RNA viral polymerases [6]. First studied during the Ebola virus epidemic but abandoned due to a lack of efficacy, its favorable safety profile in initial studies paved the way for RDV to be repurposed for COVID-19 after it demonstrated in vitro activity against severe acute respiratory syndrome coronavirus 2 (SARS-CoV-2) [7]. Studies in hospitalized adults with COVID-19 showed a potential benefit of RDV compared to placebo with respect to mortality, progression to severe disease, and time to recovery, though its efficacy was primarily limited to adults requiring minimal respiratory support and was questionable in more severely ill patients [8–10]. While adverse events (AEs) including transaminitis, anemia, hypotension, bradycardia and other arrhythmias, and diarrhea were associated with RDV in adult studies, these were typically mild and did not commonly require discontinuation of the drug [11, 12]. By October 2020, RDV became the first drug approved by the U.S. Food and Drug Administration (FDA) for the treatment of COVID-19 in hospitalized patients 12 years of age and older and weighing at least 40 kg [13].

In 2022, preliminary data from the phase II/III single-arm CARAVAN study revealed that RDV was generally well-tolerated by the 56 pediatric patients enrolled in the trial, with 85% of children demonstrating clinical improvement while on the drug [14]. Other pediatric observational studies would also go on to replicate these high rates of clinical improvement with RDV [15, 16]. Following the encouraging results of both the CARAVAN study and adult trials, RDV subsequently became the first, and only COVID-19 treatment approved by the FDA for use in children 28 days and older weighing at least 3 kg who are hospitalized or at high risk for progression to severe COVID-19 [17]. While multiple studies, including the aforementioned CARAVAN trial, have investigated the safety of RDV in children and suggest a similarly favorable adverse effect profile as seen in adults, many of these pediatric studies are limited by small cohorts [14–16, 18–20]. In the absence of a randomized controlled trial (RCT) to investigate RDV use in children, larger studies are imperative to build more robust data surrounding RDV safety in the pediatric population. Hence, we aimed to share our experience with the safety of RDV during the COVID-19 pandemic in a large number of children.

## Methods

This retrospective observational study received institutional review board approval from University of Illinois College of Medicine at Peoria and identified all patients 0–18 years old in the OSF HealthCare system who were admitted for COVID-19 and received at least one dose of RDV between March 2020 and December 2022. The OSF HealthCare system encompasses 14 hospitals in central and northern Illinois and includes the Children’s Hospital of Illinois, the largest children’s hospital in downstate Illinois.

Within the OSF HealthCare system, children could be considered for treatment with RDV if they met the indications put forth in the National Institutes of Health (NIH) COVID-19 Treatment Guidelines [21]. Namely, children could receive RDV if they were (1) SARS-CoV-2 positive by polymerase chain reaction or rapid antigen testing and (2) had severe disease (defined as SpO_2_ < 94% on room air or requiring supplemental oxygen) or at high risk for progression to severe disease. Risk factors for progression to severe disease were defined in accordance with the aforementioned COVID-19 Treatment Guidelines as the presence of ≥ 1 of the following comorbidities: cardiac disease, neurologic disorders, prematurity, diabetes, obesity, chronic lung disease, feeding tube dependence, and immunocompromised status [21]. RDV was administered intravenously over a period of 2 h at an initial dose of 200 mg with subsequent doses of 100 mg for patients ≥ 40 kg, or at an initial dose at 5 mg/kg with subsequent doses of 2.5 mg/kg for patients < 40 kg. Patients could receive up to a 5-day course of RDV depending on their clinical status; children at high risk for severe disease but otherwise asymptomatic or mildly symptomatic received up to a 3-day regimen unless extended to 5 days by clinician judgment.

Hypotension was defined per pediatric advanced life support (PALS) criteria or by receipt of fluid bolus for lower blood pressure per clinician judgment [22]. Hypertension was defined per the 2017 American Academy of Pediatrics practice guideline [23]. Bradycardia was defined per the PALS algorithm [24]. Transaminitis was defined by the 2017 Common Terminology Criteria for Adverse Events (CTCAE) [25]. Acute kidney injury was defined by the 2012 Kidney Disease Improving Global Outcomes (KDIGO) practice guideline [26]. After potential adverse events were identified by chart review, they were adjudicated based on principles outlined by Naranjo et al. [27]. If there was a probable causal relationship between RDV and the adverse event, it was assigned a CTCAE grade.

Data analysis was conducted using Stata/MP 13.1 (StataCorp, TX, USA). Quantitative variables (e.g., age, length of stay, inflammatory markers) were expressed as numbers (%), median, and interquartile range (IQR). Qualitative variables (e.g., gender, comorbidities, clinical presentation) were presented as numbers (%). Liver enzyme and creatinine levels following RDV administration were not available for 6 (9%) and 7 (11%) patients, respectively. These patients were excluded from the incidence calculations for the respective AE. The Hepatocyte Injury Index (HIX) score, which is a measure of AST and ALT after correcting for the delay in liver enzyme clearance, was used to determine the trajectory of liver injury at time of completion of RDV therapy or discharge, whichever came earlier [28]. The Chi-square test compared the frequency of AEs in those with mild-moderate COVID-19 but at high risk for disease progression versus all children with severe COVID-19, with statistical significance set at *p* < 0.05.

## Results

**Fig. 1:**
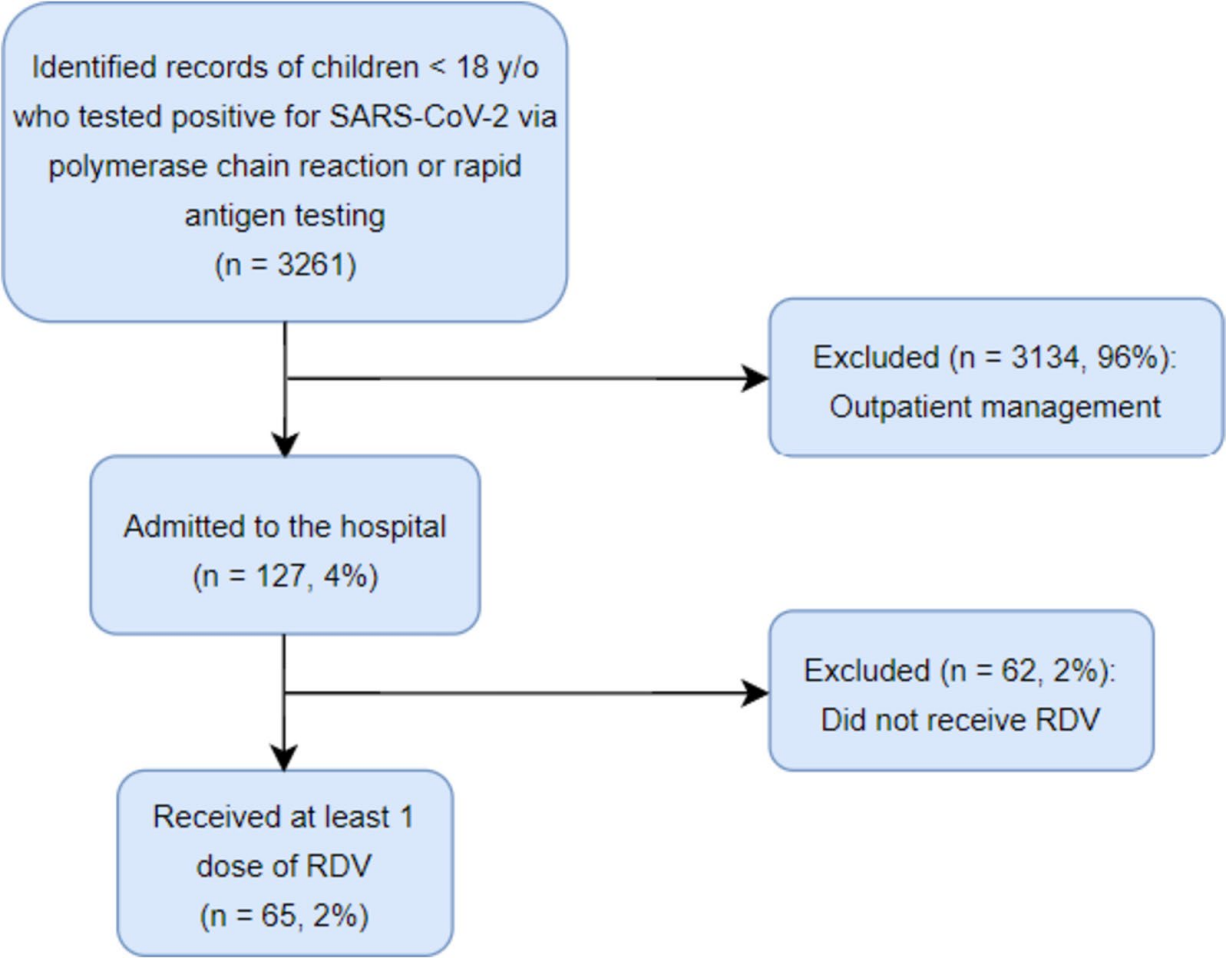
Flowchart of children screened for inclusion in the study. The parent cohort contained 3261 children who tested positive for SARS-CoV-2. 65 patients met criteria for inclusion in the sub-study. *Abbreviations: RDV = remdesivir*

A total of 3261 medical records of children ≤ 18 years old who tested positive for SARS-CoV-2 between March 2020 and December 2022 were identified (Fig. 1). Of these, 127 children were admitted to the hospital. A total of 65 hospitalized children received at least one dose of RDV, with baseline characteristics of the cohort summarized in Table 1. Admission diagnoses included pneumonia in 46 patients (71%), upper respiratory tract infection in 13 patients (20%), acute febrile illness in 5 patients (8%), and asymptomatic SARS-CoV-2 infection in 1 patient (1%). Twenty-three patients (35%) were considered “medically complex” as defined as having ≥ 3 comorbidities, and 22 children (34%) had only mild-moderate COVID-19 at presentation but were admitted for RDV infusions given their risk factors for severe illness. Details of the hospital course are outlined in Table 2. The median duration of time between symptom onset and start of RDV was 3 days. Of the patients who required supplemental oxygen during admission, the median duration of oxygen requirement after the start of RDV was 2 days. Almost half of patients (45%) did not complete their RDV course due to symptomatic improvement and hospital discharge, and all but one patient (98%) were discharged by day 23 of admission. No patients required new use of home supplemental oxygen, though three patients on chronic supplemental oxygen at baseline saw a slight increase in their home oxygen requirements at time of discharge. No patients progressed to require extracorporeal membrane oxygenation (ECMO), and no deaths of any cause occurred during hospitalization.

**Table 1:** Baseline characteristics

Overall cohort (*n* = 65)	Number, %
Male sex	41 (63)
Median age (years)	8.7 (IQR 3–15 years; range 3 months–18 years)
Patient age	
< 6 months old	3 (5)
6 months–4 years old	20 (31)
5 years old–11 years old	16 (24)
≥ 12 years old	26 (40)
Pre-existing comorbidities	
Pulmonary	20 (31)
Cardiac	4 (6)
Hematologic/oncologic	20 (31)
Neurologic	13 (20)
Chromosomal abnormality	7 (11)
Obesity	13 (20)
Patients with ≥ 3 comorbidities	23 (35)
**Mild-moderate COVID-19, high-risk for severe disease (*n*** **= 22)**	
Male sex	14 (64)
Median age (years)	8.5 (IQR years 4.5–14.5 years; range 1–17 years)
Patient age	
< 6 months old	0 (0)
6 months–4 years old	6 (27)
5 years old–11 years old	9 (41)
≥ 12 years old	7 (32)
Risk factor for severe disease	
Malignancy	13 (59)
Hematologic disorder, non-malignant)	5 (23)
Neurologic	2 (9)
Other immunocompromised state	2 (9)

**Table 2:** Characteristics of hospitalized patients

**Overall characteristics (*n*** **= 65)**	**Number, %**
Baseline oxygen requirement	
Room air	29 (45)
Low flow	32 (49)
High flow	4 (6)
Presence of co-viral infection	12 (18)
Median duration of hospital admission (days)	
Overall cohort	4 (IQR 3–5 days; range 1–48 days)
Patients with ≥ 3 comorbidities	5 (IQR 4–7 days; range 2–23 days)
Median duration of symptom onset to start of RDV (days)	3 (IQR 2–5 days; range 0–15 days)
Required PICU admission	16 (25)
Highest level of oxygen required	
Room air	22 (34)
Low flow	21 (32)
High flow	18 (28)
Mechanical ventilation	4 (6)
Median duration of oxygen support after start of RDV (days)	2 (IQR 1–5 days; range 1–44 days)
Median duration of RDV treatment (days)	2
Duration of RDV therapy (days)	
1	2 (3)
2	10 (15)
3	26 (40)
4	8 (12)
5	19 (30)
Concomitant SARS-CoV-2 therapies received	
Anticoagulation	37 (57)
Corticosteroids	53 (82)
Antibiotics	25 (38)
Baricitinib	6 (9)
Anakinra	1 (2)
**Mild/moderate COVID-19, high-risk for severe disease (*n*** **= 22)**	**Number, %**
Duration of RDV treatment (days)	
2	2 (9)
3	15 (68)
4	0 (0)
5	5 (23)
Highest level of oxygen required	
Room air	20 (91)
Low flow	2 (9)

*RDV* remdesivir, *IQR* interquartile range, *PICU* pediatric intensive care unit

Out of 65 children in the cohort, 19 (29%) experienced an AE attributed to RDV, all of which were only mild or moderate (CTCAE grade I or II) (Table 3). In the 22 children with mild-moderate COVID-19 but at high risk for disease progression, the incidence of AEs was not different than that of the children receiving RDV for severe disease (Χ^2^ (1) = 0.03, *p* = 0.8).

**Table 3:** Adverse events by CTCAE grade

**Overall cohort**	**Number, %**
Experienced ≥1 adverse event	19 (29)
Hypotension	3 (5)
Grade I	0 (0)
Grade II	3 (5)
Required fluid bolus	2 (3)
Bradycardia	5 (8)
Grade I	4 (6)
Grade II	1 (2)
Elevated AST/ALT	13 (22)
Grade I	12 (20)
Grade II	1 (2)
Ventricular arrhythmia	1 (2)
Grade I	0 (0)
Grade II	1 (2)
**Mild/moderate COVID-19, high-risk for severe disease**	**Result**
Experienced ≥1 adverse event	6 (27)

*CTCAE* common terminology criteria for adverse events, *AST* aspartate aminotransferase, *ALT* alanine aminotransferase

Transaminitis was the most commonly observed AE, present in 22% of patients. Over half of these patients had an elevated aspartate aminotransferase (AST) or alanine aminotransferase (ALT) prior to RDV administration but saw further increase after starting the drug. However, the transaminitis was overall mild; of the 13 patients with elevated AST/ALT, all but one had liver enzymes between 1.5 and 3 times the upper limit of normal (ULN), and no patient had liver enzymes greater than 5 times the ULN. Of these 13 patients with transaminitis, calculation of the HIX score indicated an improving trajectory of liver injury in 67% of patients at completion of RDV therapy or at time of discharge. Approximately 40% of patients saw complete resolution of their transaminitis prior to completing the RDV course.

Less common AEs included hypotensive episodes, which were observed in three patients (5%). All hypotensive episodes occurred during the RDV infusion, with two patients experiencing hypotension during the majority of RDV doses and one patient developing hypotension solely during the loading dose. Only minor interventions were required to correct the hypotensive episodes (i.e., fluid boluses, temporary discontinuation of home antihypertensives). Bradycardia was present in five patients (8%), none of whom had cardiac comorbidities. Apart from one patient who developed bradycardia during the first infusion of RDV, all other bradycardic episodes were delayed with relation to drug administration, with an average of 8 h (range 4–14 h) from completion of the RDV infusion to the onset of bradycardia. One patient required temporary discontinuation of their home beta-blocker. No interventions were necessary in the remaining patients. One patient with underlying structural heart disease developed premature ventricular contractions (PVCs) while on RDV; however, the arrhythmia resolved after the patient’s concurrent hypokalemia was corrected. No cases of hypertension or acute kidney injury attributable to RDV were observed. With the exception of the one patient who developed PVCs, none of the AEs led to early discontinuation of RDV.

There were five children who required hospitalization for 14 or more days, all of whom were medically complex and/or had risk factors for severe COVID-19. Two children required a prolonged stay due to COVID-19-related respiratory failure, while the remainder had their discharge delayed for management of pre-existing complex medical conditions (e.g., seizure disorder, hematological disease). Notably, there was one patient with a history significant for obesity who required long-term hospitalization (48 days). He was admitted and began RDV approximately 1 week after symptom onset but quickly declined and required intubation for mechanical ventilation. His hospital course was complicated by persistent air leak and recurrent ventilator-associated pneumonia, necessitating prolonged intubation for 36 days. During his hospitalization, the patient did develop a grade II elevation of AST and a grade I elevation of ALT, though both resolved 4 days after starting RDV and were not determined to be related to his clinical deterioration. This patient was successfully weaned to room air prior to discharge to a rehabilitation facility for reconditioning.

## Discussion

Overall, our work demonstrates that RDV appears to be a safe and well-tolerated drug in pediatric populations. While close to one-third of patients experienced at least one adverse event potentially related to RDV, no serious AEs were reported, and the AEs that did occur rarely required intervention or discontinuation of the drug. Though definitive conclusions cannot be drawn due to the observational nature of our study, we have summarized the prior pediatric RDV safety studies in Table 4, and our conclusions are bolstered by the similarly low rates of severe AEs and discontinuation of RDV across these studies.

**Table 4:** Summary of previous studies of RDV adverse effects (select) in pediatric patients

	Mendez-Echevarria et al. (2020) [20]	Goldman et al. (2021) [16]	CARAVAN study, interim results (2022) [14]	Manabe et al. (2022) [18]	Jugulete et al. (2023) [19]	Samuel et al. (2023) [15]
Methodology and study sample	Multicenter observational study of children < 16 y/o approved for compassionate-use RDV during March 2020	Multicenter observational study of children < 18 y/o approved for compassionate-use RDV from March to April 2020	Multicenter, phase 2/3 single arm, open-label study of children < 18 y/o	Single-center, retrospective observational study of patients ≤ 19 y/o with mild-severe COVID-19 from February to June 2020	Single-center, retrospective cohort study of patients < 18 y/o with mild-severe COVID-19 who received RDV vs. symptomatic treatment alone^†^ from July 2020 to September 2022	Single-center, retrospective observational study of patients < 18 y/o with severe COVID-19 from October 2020 to February 2022
Country	Spain	International (USA, Spain, UK, Italy, France, Germany)	International (USA, Spain, UK, Italy)	Japan	Romania	USA
Sample size (*n*)	8	77	53	20	126	48
Patients on invasive respiratory support at baseline	4 (50)	39 (51)	12 (23)	0	0	0
Maximum duration of RDV (days)	10	10	10	10	10	5
Adverse event, *n* (%)						
Hypotension	0	-	-	0	-	-
Hypertension	0	1 (1)	4 (8)	0	-	26 (54)
Bradycardia	0	1 (1)	3 (6)	0	-	6 (13)
Acute kidney injury	0	1 (1)	6 (11)	0	0	0
AST increased	0	4 (5)	-	4 (20)	78 (62)*	0*
ALT increased	0	5 (7)	4 (8)*	3 (15)	68 (54)*	0*
Serious AE	0	12 (16)**	0	0	0	0
Discontinued RDV	0	5	2	0	0	-

*RDV* remdesivir, *AST* aspartate aminotransferase, *ALT* alanine aminotransferase, *AE* adverse effect

*Defined as > 5 × upper limit of normal

**Majority of serious AEs were attributed to COVID-19 or comorbidities

†Study is limited by the lack of inclusion criteria to delineate which patients received RDV vs. symptomatic treatment alone

Transaminitis was by far the most commonly observed AE in our study, an observation consistent with nearly all prior investigations (Table 4). Though there is a wide range in the reported incidences of elevated AST (0–62%) and ALT (0–54%) in the literature, this is likely influenced by variations in cohort size, duration of RDV therapy, and the definition of transaminitis (i.e., liver enzymes > 1.5 × versus > 5 × ULN) utilized by each study. Due to the absence of a control group in our study, we cannot neglect a potential effect of concurrent SARS-CoV-2-induced hepatotoxicity on the incidence of transaminitis. SARS-CoV-2 has been hypothesized to cause liver injury via direct cytotoxic effects on hepatocytes, as well as through pathological inflammation arising from hypoxia and a dysregulated immune response [29]. The improving trajectory of transaminitis seen in two-thirds of our patients prior to completion of RDV therapy further suggests a viral driver of liver enzyme elevation. However, this likely does not account for all observed liver enzyme derangements, as one study examining RDV in children with COVID-19 found significantly higher rates of transaminitis in children receiving RDV compared to those who did not receive the drug, suggesting that RDV itself may contribute to elevated liver enzymes [19].

Despite the relative frequency with which transaminitis is observed, both our work and prior studies demonstrate that this rarely necessitates discontinuation of RDV. Furthermore, while our study was limited by a lack of follow-up to ensure resolution of the elevated liver enzymes, RDV-associated transaminitis has been shown to resolve spontaneously [16, 19].

Similarly, our observed incidence of bradycardia was within the range of previous studies’ reports (Table 4). RDV’s proarrhythmic effects are likely mediated by its major active metabolite and adenosine analogue (GS-443902), as well as its inhibition of mitochondrial RNA polymerases in cardiomyocytes [6, 30]. With a half-life of 11 h, GS-443902 has a markedly longer half-life than adenosine itself, which may explain the delayed nature of the bradycardic episodes seen in our patients [6]. While RDV’s proarrhythmic effects have been documented in adults, with reports of QT prolongation, atrial fibrillation, heart block, and rarely cardiac arrest, there remains a paucity of reports of RDV-induced arrhythmias beyond sinus bradycardia in the pediatric population [12]. Notably, our study identified one patient with congenital heart disease who developed PVCs, necessitating discontinuation of RDV. This patient was found to have concurrent hypokalemia, an electrolyte derangement known to be associated with ventricular arrhythmias and achieved resolution of his PVCs following potassium correction. We were unable to rule out this arrhythmia as an effect of RDV, as hypokalemia has also been occasionally documented with RDV use [18]. Additionally, the proarrhythmic effects of RDV may be amplified in individuals with structural heart disease [12]. Arrhythmias in the pediatric population appear to be an uncommon AE overall; however, close monitoring of electrolytes and continuous telemetry while on RDV may be prudent, particularly in children with underlying structural heart abnormalities.

While acute kidney injury was one of the most frequently noted AEs in the preliminary results of the CARAVAN study with 11% of patients developing renal impairment, this finding could not be replicated by either this study or prior investigations in children (Table 4) [14]. As the CARAVAN study enrolled a higher proportion of patients with critical COVID-19, this finding may be a consequence of severe illness rather than drug-induced renal impairment [31]. Furthermore, a meta-analysis of adult RCTs similarly failed to demonstrate an increased incidence of acute kidney injury in patients receiving RDV, suggesting that renal damage is more likely a function of SARS-CoV-2 infection itself [32]. Together with previous studies, our work contributes to the growing evidence arguing against a nephrotoxic effect of RDV in pediatric populations.

Approximately one-third of the patients in our study had only mild or moderate COVID-19 but were admitted for RDV therapy due to their high risk of progression to severe disease from underlying comorbidities (e.g., malignancy, sickle cell anemia). Currently, FDA support for this specific indication in children is largely based on a single RCT that assessed the impact of a 3-day RDV regimen on COVID-19 progression in high-risk patients ≥ 12 years old in the outpatient setting, despite only enrolling eight patients under the age of 18 [33]. While the FDA cited favorable results from the aforementioned pediatric safety studies as additional support for this indication, these studies did not specifically report on the safety of RDV use in high-risk children with only mild-moderate COVID-19. To our knowledge, this is the first study to provide data regarding AEs associated with RDV use in this specific population, demonstrating a similar incidence of AEs between this high-risk subgroup and the remaining cohort with severe COVID-19. While unable to assess the efficacy of RDV therapy due to the observational design of the study, given that only two out of 22 high-risk patients progressed to require oxygen therapy suggests that RDV may have value in preventing severe disease in children.

While COVID-19 in infants is generally mild and rarely requires hospitalization, there is still a need for safe and effective COVID-19 therapies in this age group given the lack of an FDA-approved COVID-19 vaccine for children under the age of 6 months [34]. While the previously cited safety studies have enrolled children as young as 1 month old, few have specifically reported the incidence of AEs in this age group. Three children under 6 months old who received RDV were included in our cohort, none of whom developed an AE. This finding, coupled with the absence of AEs observed in any child under the age of 2 in our study, further bolsters confidence in the safety of RDV in our youngest patients.

Our study has several advantages, the first of which is that it is one of the largest investigations into the safety of RDV in pediatric populations to date. In contrast to studies with a significant proportion of patients already requiring mechanical ventilation prior to RDV, such as the CARAVAN trial, the lower baseline severity of patients in our study makes it more generalizable to the average pediatric population, in which children requiring invasive respiratory support comprise only a small proportion of hospitalized patients [34]. Limitations of our study include a reliance on retrospective chart review. It is possible that minor AEs (i.e., nausea, rash, headache) were not documented in the chart and thus not identified by this study. Despite being one of the larger safety studies of RDV in children to date, our sample size of 65 is still relatively small and may have prevented us from identifying rare but potentially life-threatening adverse effects. The lack of a control group also limits us in our attribution of an AE to RDV versus COVID-19 itself, co-viral infection, or underlying comorbidities, and it prevents us from completely ruling out effects of concomitant medications and other COVID-19 therapies (i.e., corticosteroids, baricitinib).

## Conclusions

Our retrospective study of RDV in children with COVID-19 demonstrates the safety of this pharmacotherapy in the pediatric population, as all AEs were either mild or moderate and rarely necessitated discontinuation of the drug. The most commonly observed AE was transaminitis, followed by bradycardia and hypotension. A similarly favorable adverse effect profile was seen in high-risk children with mild-moderate COVID-19. Rates of clinical improvement in the overall cohort at one month were extraordinarily high, and very few high-risk patients progressed to require supplemental oxygen once started on RDV. While placebo-controlled trials are warranted to establish definite conclusions about its safety and efficacy, our study provides a valuable contribution to the growing literature supporting the safety and tolerability of RDV in pediatric patients with COVID-19.

## Data Availability

No datasets were generated or analysed during the current study.
